# Ethnobotanical survey of cooling herbal drinks from southern China

**DOI:** 10.1186/1746-4269-9-82

**Published:** 2013-12-19

**Authors:** Yujing Liu, Selena Ahmed, Chunlin Long

**Affiliations:** 1 College of Life and Environmental Sciences, Minzu University of China, Beijing 100081, China; 2 Department of Health and Human Development, Montana State University, Bozeman, MT 59717, USA; 3 Kunming Institute of Botany, Chinese Academy of Sciences, Kunming 650201, China

**Keywords:** *liáng chá*, Cooling tea, Ethnomedicine, Botanical industry

## Abstract

**Background:**

*Liáng chá* (“cooling tea”, “herbal tea” or “cool tisane” in Chinese) are herbal drinks widely produced in southern China and consumed by billions of people worldwide to prevent and treat internal heat as well as a range of associated health conditions. Globalization and renewed interest in botanical remedies has attracted growing attention in cooling herbal drinks by industry, scientists and consumers. However, there is a knowledge gap on the plant species used and commercialized for cooling herbal drinks in southern China and their associated ethnobotanical use, habitat and conservation status. This is the first study to document plant species used and commercialized as *liáng chá* in southern China’s Lingnan region and associated ethnomedical function, preparation methods, habitat and conservation status.

**Methods:**

Three hundred market surveys were conducted between 2010-2012 in the largest herbal drink producing region of China to record plants used for *liáng chá* and to document knowledge on their medicinal function, habitat and conservation status. Product samples and voucher specimens were collected for taxonomic identification.

**Results:**

All informants harvest and cultivate plants for preparing herbal drinks for their medicinal, cultural and economic values. A total of 222 ethnotaxa corresponded to 238 botanical taxa (species, varieties or subspecies) belonging to 86 families and 209 genera were recorded as *liáng chá* to treat health conditions in the study area. Recorded remedies consisted of one or several plant species to treat conditions classified into 27 major health conditions with clearing internal heat being the most common medicinal function. The habitat types of plants documented for use as *liáng chá* include 112 wild harvested species, 51 species that are either wild harvested or cultivated, 57 cultivated species, and 2 naturalized species. According to China’s Red List and CITES on conservation status, one of these species is endangered, one species is critically endangered, eight species are vulnerable, one is listed in CITES II, three are listed in Regional Red Data Book and the remaining 224 species are in the least concerned conservation category.

**Conclusions:**

The *liáng chá* industry of southern China reflects the plant species richness and cultural diversity of the region. Future research on safety and efficacy of herbal drinks as well as ecological and cultural conservation efforts are needed for the sustainable growth of China’s botanical industry.

## Background

**Figure 1:**
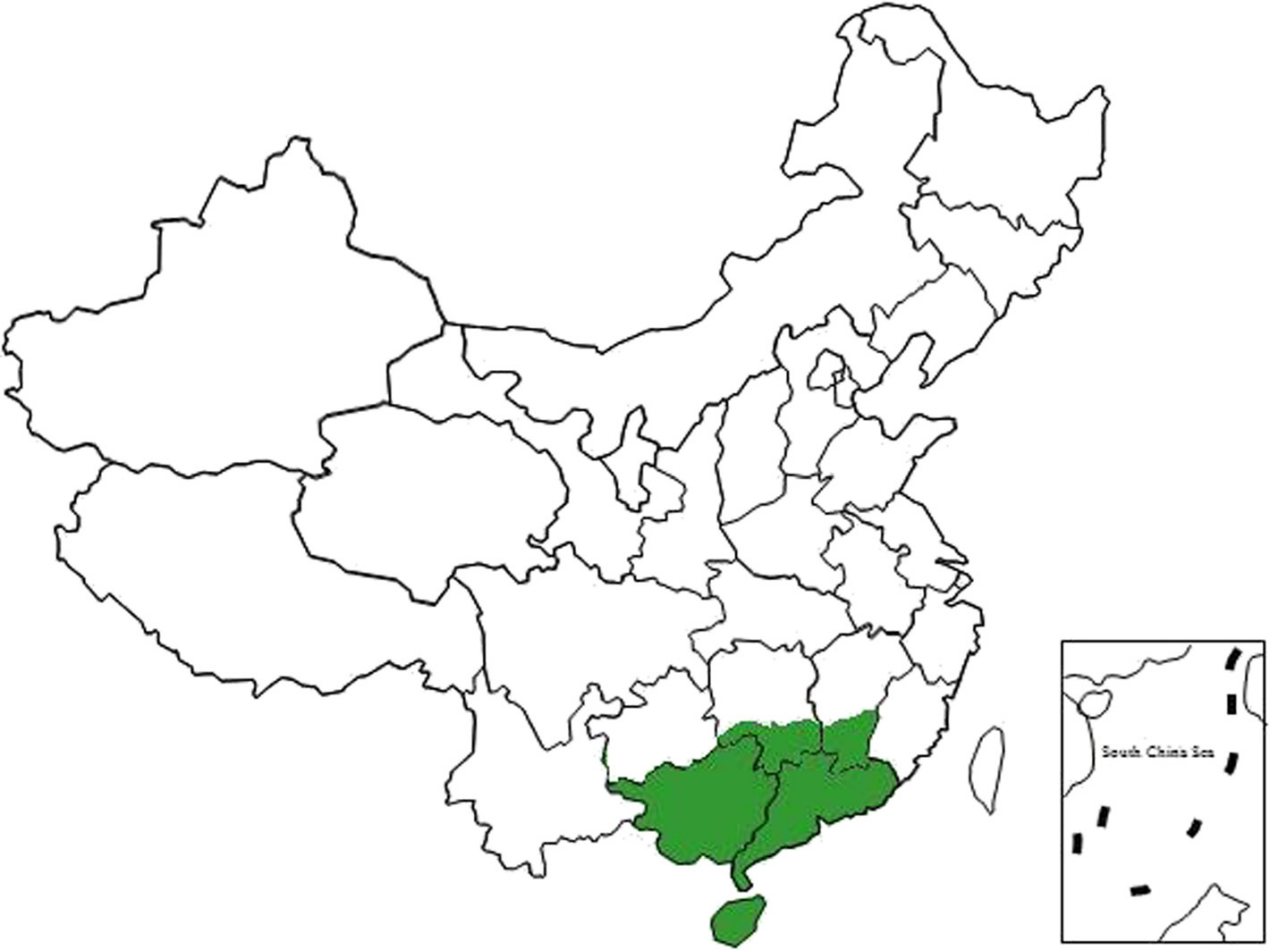
Location map of study site.

*Liáng chá* refers to “cooling tea” or herbal tisanes/teas produced from water infusions of a range of plant species other than the caffeine-containing tea plant (*Camellia sinensis*; Theaceae). Plant material may consist of fresh or dried leaves, fruit, flowers, pollen, nuts, barks, seeds and roots from a single species or multiple species. The practice of drinking *liáng chá* is an ancient custom regarded to have originated from the Lingnan region of southern China over 2,000 years ago. Lingnan is the tropical and subtropical region south of China’s Nanling Mountains that covers all or part of Guangdong, Guangxi, Hainan, Hong Kong, Macau, Hunan and Jiangxi provinces (Figure 1). This region is notable for its rich biodiversity and cultural practices.

The damp humidity and heat levels of the Lingnan region are thought to contribute to a health condition in local human populations recognized as “internal heat”. According to traditional Chinese medicine as well as various ethnomedical systems of China’s minority socio-linguistic groups, high temperature inside the human body above 37°C causes internal heat that may result in discomfort and a range of health conditions. In particular, humidity and temperature transition during the month of March is recognized as a time when local communities are particularly susceptible to the illnesses caused by internal heat such as influenza, fever, pneumonia and cough. In addition to humidity and temperatures, several other factors are considered to contribute to internal heat including spicy food, irregular work and rest patterns, and emotional dissonance. Local populations collected herbs from surrounding mountains and valleys and prepared these in hot water and consumed them at room temperature to reduce internal heat and to treat associated health conditions.

The rich biocultural diversity of Lingnan has resulted in a practice of producing and consuming cooling herbal drinks following the framework of traditional Chinese medicine and various ethnomedical systems of China’s minority socio-linguistic groups. While many botanical products were eliminated as ineffective or replaced by synthetic products during the early 20th century [1], the resurgence of interest in natural products during the past 35 years has fueled the global herbal tisane market. Approximately 25% of the drugs prescribed worldwide come from plants, specifically from 121 natural active compounds with 252 natural product drugs that the World Health Organization (WHO) considers as essential [2]. WHO estimates that approximately 75-80% of the world’s population relies on indigenous or traditional plant medicines either in part or entirely for their primary health care needs [3]. In particular, herbal remedies remain the cornerstone of treatment in underdeveloped regions and among the aboriginal peoples of the world [2,4] while increasingly becoming part of a globalized health supplement market. Most of this therapy involves the use of plant extracts, often in aqueous solutions [3]. Numerous studies have validated that the medicinal properties of herbal drinks derive from their phytochemical profiles [1,5].

Despite the renewed interest in botanical remedies by scientists, consumers, and industry, there remains a knowledge gap on the plant species used and commercialized for cooling herbal teas in southern China and their associated habitat and ethnobotanical use. This is the first study to document plant species used and commercialized as *liáng chá* in southern China’s Lingnan region and associated habitat and ethnomedicinal knowledge. The plant material, medicinal uses, part used, habitat and safety of herbal drinks known as *liáng chá* were recorded for informing future investigation, practice and policy.

## Methods

### Study sites

Lingnan is the tropical and subtropical region south of China’s Nanling Mountains. It covers an area of 450,000 km^2^ in Guangdong, Guangxi and Hainan provinces as well as parts of Hunan and Jiangxi provinces. Research was conducted in Guangdong, Guangxi, Jiangxi, Hainan and Hunan provinces of the Lingnan region. The region has a high population of 160 million. The geological and topographic variety of the region has contributed to rich biodiversity.

### Ethnobotanical data collection and statistic analysis

Field surveys were conducted between 2010 and 2012. Special care was taken in choosing the informants. Only those who were born and have always been living in Lingnan region were taken into consideration. A total of 300 informants were interviewed including 148 males and 152 females between the ages of 35-70 from 12 socio-linguistic groups. They are from Hakka, Cantonese, Zhuang, Dong, Li, Shui, Molao, Maonan, Yao, Miao, Gelao, and Han Chinese. In each group we interviewed at least 15 informants, while more than 35 informants from each major group (Cantonese, Hakka, Han Chinese, or Zhuang) were visited and interviewed. Approximately 60 informants of equal age and gender groups were interviewed in every study province. As for educational background, 44 had never gone to school at all, 163 had only a primary-school education, 31 had a grammar-school education, 36 had a high-school education, and 26 had university degrees. Surveys consisted of semi-structured questionnaires, open-ended interviews and participatory observation. The survey instruments included questions on plants used for *liáng chá* and their associated habitat, medicinal functions and preparation. Interviews were conducted in standarded Chinese (*putonghua*) and informant responses were recorded using a digital voice recorder and transcribed. Product samples were collected from markets, mountains, forests and farming fields. Key informants were selected to guide collections of voucher specimens in the surrounding mountains and fields. Photographs were taken to record all plant species and gathering activities. Voucher specimens of all plants available during field investigations were collected and deposited in the herbarium of Minzu University of China. Identification of voucher specimens was followed the *Flora of China* and the collections in PE (the Herbarium, Institute of Botany, Chinese Academy of Sciences), IBSC (South China Botanical Garden Herbarium, Chinese Academy of Sciences) as well as KUN (the Herbarium, Kunming Institute of Botany, Chinese Academy of Sciences). The folk names were not recorded, because the writing characters of Hakka, Cantonense and Han Chinese were the same although their pronunciation is different.

## Results and discussion

### Plant material for cooling herbal tisanes

**Figure 2:**
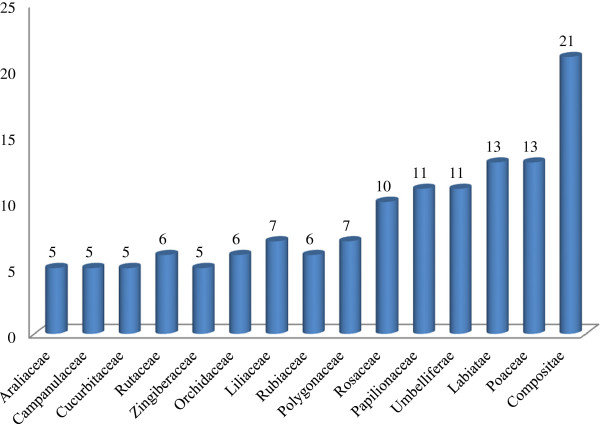
Most quoted plant families in %, f (the number of species included in family) > 5, (f < 5 are summarized as “others”) (n = 84).

**Figure 3:**
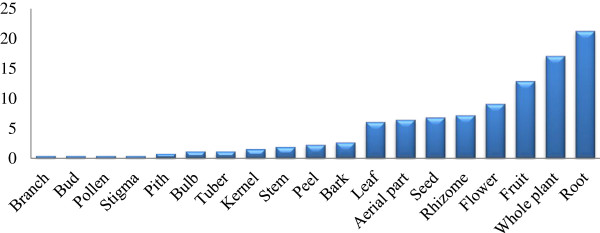
Plant parts used as herbal drinks.

A total of 238 species from 86 families and 209 genera were recorded for use in *liáng chá*, or cooling herbal tisanes, or herbal drinks, in the Lingnan region. Some of them are weeds, in traditional medicinal floras has been overlooked, because widely utilized medicinal plants need to be abundant and accessible, rare plants are not often found in medicinal floras [6]. The most prevalent botanical family represented in the collections was the Asteraceae with 21 species followed by Poaceae with 13 species and Lamiaceae with 12 species (Figure 2). The documented cooling herbal drinks consisted of a range of plant parts including leaf, root, fruit, flower, branch, bud, pollen, stigma, pith, bulb, tuber, kernel, stem, peel, aerial part, seed, bark and rhizome from herbaceous and woody plants. The root was the most common plant part used for making *liáng chá* and accounted for 56 of the 238 species, or 21.3% (Figure 3). Decoction in hot water was the primary used method for preparing *liáng chá*. A vast majority (>80%) of preparations involved drying plants before decoction in order to preserve herbs and their properties as well as to improve the taste. Only 26 of the 238 species used for *liáng chá* were specified as being from either fresh or dry plant material. Six species were specified as needing to be processed before decoction and only one species, *Glycine max,* is fermented before consumption (See Additional file 1: Table S1). A total of 112 species used for *liáng chá* are wild harvested, 57 species are cultivated, 51 species are either wild harvested or cultivated, and 2 are natural domestication. Wild harvested species were collected in montane forests, wetlands, shrub lands, and disturbed habitat (See Additional file 2: Table S2).

Prof. Shiu-ying Hu recorded 66 herbal tea species according to parcelled herbal tea in Hong Kong stores of USA and field work in Hong Kong [7]. Compared to Hong Kong herbal tea, there are similarities with Lingnan herbal tea. For example, Chinese foxglove (di-huang, 地黄, *Rehmannia glutinosa*), jade bamboo (Yu-zhu, 玉竹, *Polygonatum odoratum*), Eucommia (du-zhong, 杜仲, *Eucommia ulmoides*) and so on. However, there are some differences between two regions. For instance, *Youngia japonica* (Huan-yang-cao, 还阳草), *Solanum torvum* (Shui-qie, 水茄), *Polygonum perfoliatum* (Lao-hu-ci, 老虎刺) and some others were not used in Lingnan region.

### Medicinal functions of cooling herbal tisanes

**Figure 4:**
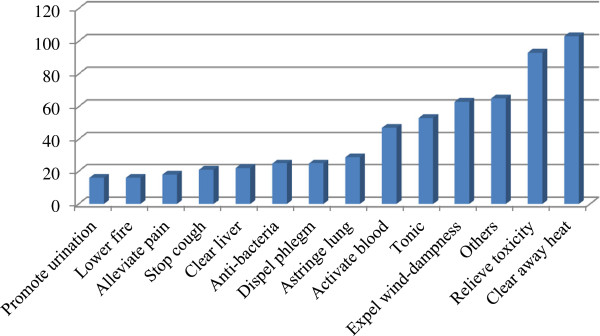
Ailment category--frequency of mentions within one ailment > 10 (f < 10 are summarized as “others”).

Herbal teas are no limit to the duration that herbal teas are consumed as they are used within a food context, not for the treatment of medical conditions [8]. Here, the medicinal functions of recorded herbal drinks can be divided into 27 health conditions (Figure 4). The most common function is clearing away heat, followed by detoxification. In addition to the 13 types of medical functions shown in Figure 4, there are 14 other functions including reducing blood pressure, warming lung and dispersing cold, warming spleen and stomach, resolving macula, anti-tumor, relaxing the bowels, alleviating edema, promoting lactation, relieving thirst, sedative, improving vision, promoting digestion, stopping vomiting and dysentery, and resolving sore throat. Informants were knowledgeable about the use of various herbal drinks for the prevention and treatment of different health conditions. Some of the preparations for *liáng chá* involve single plant species and others combinations of plants. In addition, some plants have a single medical function while others have multiple medical functions. A brief description of medical functions of all recorded plant species is listed as supplemental data in Additional file 1: Table S1. The medical functions that herbal teas are used for in Lingnan have evolved in response to global environmental changes and lifestyle changes driving shifts in illnesses prevalence and the emergence of new illnesses.

### The cultural importance of herbal drinks

All informants harvest and cultivate plants for preparing *liáng chá* for their medicinal, cultural and economic values. With the expanded commercialization of the botanical industry, the production of herbal teas has become an essential element of livelihood strategies of informants at the study sites. The consumption of herbal drinks is part of a regional culture and lifestyle in Lingnan. Visitors to Lingnan area are quickly introduced to the practice of drinking cooling herbal teas as a caffeine-free alternative to coffee and tea as well as a healthy alternative to sugared soft drinks. Locals of Lingnan who leave their hometown region often cherish the memory of different herbal drinks as a central part of their local culture that has been practiced for centuries.

### Commercialization of herbal tisanes

The *liáng chá* industry has dramatically grown around Lingnan to meet regional, national and global demand for herbal tea and dietary supplements. In 2010, the sales volume of Chinese herbal drinks reached 2.5 billion tons, which topped the sales volume of Coca-Cola worldwide (http://epaper.nfdaily.cn/html/2011-07/14/content_6986523.htm). China exported about one third of its herbal drinks, with the United States as the biggest importer. The popularity of herbal drinks began to acquire market demand in the United States in 1985 [1,9,10]. Herbal drink consumption has been increasing at an annual rate of 15-20%.

China has several famous *liáng chá* enterprises including Wang Laoji, Jia Duobao, He Qizheng, Wa Haha, Huang Zhenlong, Jin Hulu, Pan Gaoshou and Bai Yunshan. Wang Laoji is regarded the top *liáng chá* company for its market share and as the first commercialized recognized brand in the region. Wang Zebang, the founder of Wang Laoji, is regarded as the master of *liáng chá*. He acquired knowledge of producing cooling herbal tisanes by his family practice of hundreds of years. This herbal product is widely used for clearing summer heat and dampness as well as for detoxification. It has been so widely commercialized that it was on the menu for Kentucky Fried Chicken in China in July 2004.

Herbal drink recipes are experiencing a shift with producers changing traditional practices to meet the market demands. For example, producers and distributors have started to add sweeteners to ease the bitter flavor of herbal drinks that adds calories and change their health value. Market-monitoring efforts are imperative to protect the traditional *liáng chá* culture of Lingnan.

### Is it safe to drink cooling herbal drinks?

There are increasing studies on the safety [11-16] and pharmacological efficacy [17-21] of herbal drinks with the revitalized interest in botanicals, but most of evidence remains anecdotal based on traditional ethnomedical practice that has not been validated by controlled studies [11-14,17,18,22-26]. Findings have elucidated that many phytochemicals in *liáng chá* are beneficial to human health [17,24,26-29] while others are found to be harmful to humans [10,23,30-33]. With the growing *liáng chá* market, further research is needed to document the bioactivity and toxicity of cooling herbal drinks and their constituents by *in vitro* and *in vivo* studies. In addition, uniform standards of practice and licensing of herbal practitioners and vendors is required towards a safer *liáng chá* market. As new harvesters enter the market, it is very important for them to have the knowledge to select the proper plants as some herbs are hard to identify for their similar morphological characteristics. China’s QS certification inspection system that monitors quality throughout all production stages is one scheme towards a safer *liáng chá* market.

### Sustainability of herbal tisanes

The sustainability of the herbal drinks’ ethnomedicinal base is threatened with global environmental change, expanded commercialization, policies and overharvesting of natural resources. China’s Cultural Revolution (1966-1976) seriously damaged *liáng chá* culture by prohibiting the production and destroying associated materials.

As for the plant species used in herbal drinks, we checked the status following the China’s Red List of Higher Plants (See Additional file 2: Table S2) and CITES. Some species are involved, according to the IUCN evaluation criteria: (1) *Paris polyphylla* var. *yunnanensis* is listed as EN (endangered); (2) *Dendrobium officinale* is CR (critically endangered); (3) eight species are VU (vulnerable), including *Atractylodes macrocephala*, a few species of *Dendrobium*, *Coptis chinensis*, *Notopterygium incisum*; (4) *Nervilia fordii* and all *Dendrobium* species are listed in CITES Appendix II; (5) *Glycyrrhiza uralensis*, *Paeonia lactiflora* and *P. veitchi* are listed in Regional Red Data Book; and (6) the remaining 227 species are in the least concerned category.

Wild plant gathering is an essential element in livelihood strategies all over the world. Gathering plant has altered from one of necessity in the past to a pleasurable activity today. Wild plant gathering has therefore also received renewed attention as a form of intangible cultural heritage [34]. Chinese policy is working to protect some of its national *liáng chá* heritage. In June 2006, the state officially recognized 18 brands and 54 secret recipes of *liáng chá* under the Catalogue of National Intangible Cultural Heritage after being inherited primarily orally for over 2,000 years. However, protection is not given to the full range of herbal drinks in China and policies are needed to protect these botanical resources.

## Conclusions

The *liáng chá* industry of southern China reflects the rich plant species richness as well as cultural diversity and exchange of the region. As China’s herbal drink industry exceeds the market share of Coca-cola, future research is needed to understand the safety and efficacy of recorded herbal tisanes. The market-orientated production of herbal drinks should be monitored for ecological viability and product safety towards sustainability.

## Competing interests

The authors declare that they have no competing interests.

## Authors’ contributions

YJL and CLL conceived of and designed the study as well as conducted data collection and literature review. YJL, SA and CLL interpreted data and wrote the manuscript. All authors read and approved the final manuscript.

## Supplementary Material

Additional file 1: Table S1 Inventory of plants used in cooling herbal drink in Lingnan region of southern China. Click here for file

Additional file 2: Table S2 Habitat information and conservation need of plants used for cooling herbal drinks in Lingnan region of southern China. Click here for file
