# Supplementary material for: Ethnobotanical survey of cooling herbal drinks from southern China
Source: J Ethnobiol Ethnomed. 2013 Dec 19;9:82. doi: 10.1186/1746-4269-9-82 (PMC3926322; doi:10.1186/1746-4269-9-82)
Supplement: Additional file 1: Table S1 — Inventory of plants used in cooling herbal drink in Lingnan region of southern China. [file 1746-4269-9-82-S1.doc]

**Additional file 1: Table S1. Inventory of plants used in cooling herbal drink in Lingnan region of southern China**

(Ranked alphabetically, family names followed by generic and species names. Ethnotaxa numbered.)

| No. | Family name | Scientific name | Medical use(s) | Part of plant | Preparation before decoction |
| --- | --- | --- | --- | --- | --- |
| 1 | Acanthaceae | *Strobilanthes cusia* (Nees) J.B. Imlay | Clear heat and relieve toxicity, cool blood and relieve sore throat | Root | Dry |
| 2 | Acanthaceae | *Dicliptera chinensis* Roem. & Schult. | Clear heat and relieve toxicity, promote urination | Whole plant | Dry |
| 3 | Acanthaceae | *Rungia pectinata* Nees | Clear liver, promote diuresis to drain dampness and relieve dyspepsia | Whole plant | Dry |
| 4 | Acoraceae | *Acorus tatarinowii* Schott | Eliminate dampness and stimulate appetite, regain consciousness through dispelling phlegm, induce resuscitation and strengthen intelligence | Rhizome | Dry or fresh |
| 5 | Alismataceae | *Alisma orientale* (Sam.) Juz. | Promote dieresis to drain dampness, expel heat | Tuber | Dry or fresh |
| 6 | Alliaceae | *Allium macrostemon* Bunge | Active *yang* and dissipate accumulation, promote *qi* flow to remove stagnation | Whole plant | Dry |
| 7 | Alliaceae | *Allium sativum* L. | Warm spleen and stomach, relieve toxicity, destroy parasites | Whole plant | Fresh |
| 8 | Amaranthaceae | *Achyranthes aspera* Duss | Clear heat and purge fire | Root | Dry |
| 9 | Amaranthaceae | *Achyranthes bidentata* Blume | Activate blood and remove blood stasis, nourish the liver and the kidney, strengthen bones and muscles, alleviate edema and relieve stranguria, conduct blood-fire to go downward | Root | Dry |
| 10 | Amaranthaceae | *Amaranthus spinosus* L. | Clear heat and purge fire, eliminate toxic materialsand promote diuresis to drain dampness | Young aerial part | Dry |
| 11 | Amaranthaceae | *Celosia cristata* L. | Hemostasis, anti-bacteria | Flower | Dry or fresh |
| 12 | Apiaceae | *Angelica biserrata* (R.H. Shan & Yuan) C.Q. Yuan & R.H. Shan | Expel wind and dampness, stop pain and disperse the exogenous evil | Root | Dry |
| 13 | Apiaceae | *Angelica dahurica* (Hoffm.) Maxim. | Expel wind-dampness and stop pain | Root | Dry |
| 14 | Apiaceae | *Angelica sinensis* (Oliv.) Diels | Tonify blood, activate blood, regulate menstruation to relieve pain, moisten the bowels to relieve constipation | Root | Dry |
| 15 | Apiaceae | *Bupleurum chinense* Franch. | Release heat, soothe liver and relieve depression | Aerial part | Dry |
| 16 | Apiaceae | *Centella asiatica* (L.) Urb. | Anti-virus, anti-bacteria, reducing blood pressure | Whole plant | Dry |
| 17 | Apiaceae | *Daucus carota* var. *sativa* Hoffmann | Promote *qi* flow and soothe the middle, Promote digestion and relieve dyspepsia | Root | Dry or fresh |
| 18 | Apiaceae | *Foeniculum vulgare* Hill | Anti-bacteria | Seed | Dry |
| 19 | Apiaceae | *Ligusticum sinense* Oliv. | Activate blood and move *qi*, expel wind and alleviate pain | Root | Dry or fresh |
| 20 | Apiaceae | *Notopterygium incisum* Ting ex H.T. Chang | dispel wind-dampness | Root | Dry |
| 21 | Apiaceae | *Peucedanum decursivum* Maxim. | Direct *qi* downward and resolve phlegm, disperse wind-heat | Root | Dry |
| 22 | Apiaceae | *Saposhnikovia divaricata* (Turcz.) Schischk. | Relieve exterior and expel wind-dampness | Whole plant | Dry |
| 23 | Apocynaceae | *Plumeria rubra* L. | Clear heat and dampness, smooth lung and stop cough, eliminate toxic materials | Flower | Dry |
| 24 | Apocynaceae | *Trachelospermum jasminoides* Lem. | Clear heat and relieve toxicity | Aerial part | Dry |
| 25 | Aquifoliaceae | *Ilex asprella* Champ. ex Benth. | Clearing away heat and toxic, help produce saliva and slake thirst, detumescence | Root | Dry |
| 26 | Aquifoliaceae | *Ilex kaushue* S.Y. Hu | Clear heat and expel wind-dampness, anti-inflammatory and eliminate toxic materials | Leaf | Dry |
| 27 | Aquifoliaceae | *Ilex pubescens* Hook. & Arn. | Anti-virus | Root, leaf | Dry |
| 28 | Aquifoliaceae | *Ilex rotunda* Colebr. ex Wall. | Clear heat and promote diuresis to drain dampness, anti-inflammatory and stop pain | Bark | Dry |
| 29 | Araceae | *Pinellia ternata* Druce | Dry dampness and resolve phlegm, suppress adverse rise of *qi* to stop vomiting, relieve stuffiness and dissipate nodulation, relieve swelling and pain | Tuber | Processed with ginger |
| 30 | Araliaceae | *Panax notoginseng* (Burkill) F.H. Chen ex C.Y. Wu & K.M. Feng | Remove blood stasis to stop bleeding, promote blood circulation and alleviate pain | Root, flower | Dry |
| 31 | Araliaceae | *Panax quinquefolius* L. | Tonify *qi* and nourish *yin*, clear heat and promote fluid production | Root | Dry |
| 32 | Araliaceae | *Parthenocissus tricuspidata* Planch. | Clear away heat and promote dieresis | Root, stem | Dry |
| 33 | Araliaceae | *Schefflera bodinieri* Rehder | Cool blood and eliminate toxic materials, expel wind and dampness | Leaf, bark | Dry |
| 34 | Araliaceae | *Tetrapanax papyrifer* K. Koch | Clear away heat and promote dieresis, promote lactation | Pith | Dry |
| 35 | Asparagaceae | *Asparagus cochinchinensis* (Lour.) Merr. | Antibiosis, anti-virus, dispel phlegm and arrest cough | Root | Dry |
| 36 | Asteraceae | *Arctium lappa* L. | Relieve sore throat and resolve phlegm | Root | Dry or processed |
| 37 | Asteraceae | *Artemisia annua* L. | Clear deficiency-heat, cool blood, cure bone-steaming, relieve summer-heat and check malaria | Whole plant | Dry |
| 38 | Asteraceae | *Artemisia argyi* H. Lév. & Vaniot | Stop bleeding by warming meridians, expel cold and alleviate pain | Aerial part | Dry or fresh |
| 39 | Asteraceae | *Artemisia scoparia* Waldst. & Kit. | Clear away heat, eliminate dampness, drain the gallbladder to relieve meridians | Aerial part | Dry |
| 40 | Asteraceae | *Atractylodes lancea* DC. | Dry dampness and invigorate the spleen, dispel wind-damp, disperse cold and release the exterior | Root | Dry |
| 41 | Asteraceae | *Atractylodes macrocephala* Koidz. | Invigorate spleen and replenish *qi*, dry dampness and induce dieresis, stop sweating, prevent abortion | Rhizome | Dry |
| 42 | Asteraceae | *Carthamus tinctorius* L. | Promote blood circulation to promote menstruation, move blood stasis and alleviate pain | Flower | Dry |
| 43 | Asteraceae | *Chrysanthemum indicum* L.,  *C. lavandulaefolium* (Fischer ex Trautvetter) Makino | Clear heat and relieve toxicity, clear liver and improve vision | Flower | Dry |
| 44 | Asteraceae | *Chrysanthemum morifolium* (Ramat.) Hemsl. | Clear heat and relieve toxicity | Flower | Dry |
| 45 | Asteraceae | *Cirsium japonicum* DC. | Cool the blood and stop bleeding, eliminate toxic materials to treat carbuncle | Aerial part, root | Dry |
| 46 | Asteraceae | *Cirsium arvense* var. *integrifolium* C. Wimm. et Grabowski | Clear heat and relieve toxicity, cool blood and promote urination | Aerial part | Dry |
| 47 | Asteraceae | *Eupatorium fortunei* Turcz. | Resolve damp and clear summer-heat | Aerial part | Dry |
| 48 | Asteraceae | *Elephantopus scaber* L. | Clear heat and relieve toxicity, anti-virus | Rhizome | Dry |
| 49 | Asteraceae | *Emilia sonchifolia* Benth. | Anti-bacteria, anti-inflammation | Whole plant | Dry |
| 50 | Asteraceae | *Pseudognaphalium affine* (D. Don) Anderb. | Warm lung and replenish *qi*, dispel phlegm and arrest cough | Aerial part | Dry |
| 51 | Asteraceae | *Spilanthes acmella* (L.) L. | Resolve blood-stasis, relieve swelling and stop pain | Whole plant | Dry |
| 52 | Asteraceae | *Siegesbeckia orientalis* L. | Expel wind and dampness, dredge the meridians and collaterals | Aerial part | Dry |
| 53 | Asteraceae | *Taraxacum officinale* F.H. Wigg. | Clear heat and relieve toxicity | Whole plant | Dry |
| 54 | Asteraceae | *Tussilago farfara* L. | Moisten lung, resolve phlegm and relieve cough | Flower | Dry |
| 55 | Asteraceae | *Xanthium strumarium* Elliott | Regain consciousness | Seed | Dry |
| 56 | Berberidaceae | *Mahonia fortunei* Fedde | Nourish yin and clear heat, warm lung and stop cough | Leaf | Dry |
| 57 | Bignoniaceae | *Oroxylum indicum* (L.) Benth. ex Kurz | Clear lung and soothe throat | Seed | Dry |
| 58 | Bombacaceae | *Bombax ceiba* Burm. f. | Clear heat and relieve toxicity, promote diuresis to drain dampness and stop blood | Flower | Dry |
| 59 | Boraginaceae | *Lithospermum erythrorhizon* Siebold & Zucc. | Cool blood, activate blood, relieve toxicity and promote eruption | Root | Dry |
| 60 | Brassicaceae | *Brassica juncea* (L.) Hook. f. & Thomson | Promote urination and stop blood, clear heat and anti-inflammatory | Whole plant | Fresh |
| 61 | Brassicaceae | *Isatis tinctoria* L. | Clear heat and relieve toxicity, cool blood and resolve macula | Aerial part | Dry |
| 62 | Brassicaceae | *Raphanus sativus* L. | Promote digestion and relieve dyspepsia, direct *qi* downward and resolve phlegm | Seed | Dry |
| 63 | Burseraceae | *Canarium album* Blanco | Dry dampness, warm the middle, eliminate phlegm and check malaria | Fruit | Processed with sugar |
| 64 | Campanulaceae | *Adenophora stricta* Miq. | Nourish *yin* and smooth lung | Root | Dry |
| 65 | Campanulaceae | *Codonopsis pilosula* Nannf. | Invigorate the middle energizer and replenish *qi*, invigorate spleen and lung, nourish blood and promote fluid production | Root | Dry |
| 66 | Campanulaceae | *Platycodon grandiflorus* DC. | Disperse lung *qi* and resolve phlegm, relieve sore throat and drain pus | Root | Dry |
| 67 | Cannabiaceae | *Cannabis sativa* L. | Nourish *yin*, smooth lung and relieve sore throat | Seed | Dry |
| 68 | Caprifoliaceae | *Lonicera confusa* DC.,  *L. hypoglauca* Miq.,  *L. japonica* Thunb. | Clear heat and relieve toxicity, disperse wind and discharge heat | Flower, stem | Dry |
| 69 | Caryophyllaceae | *Pseudostellaria heterophylla* (Miq.) Pax | Tonify spleen *qi*, promote fluid production and nourish lung | Root | Dry |
| 70 | Caryophyllaceae | *Vaccaria hispanica* (Mill.) Rauschert | Activate blood and open meridians, promote lactation and cure abscess, relieve stranguria | Seed | Dry |
| 71 | Combretaceae | *Quisqualis indica* Blanco | Destroy parasites, remove food stagnation | Seed | Dry |
| 72 | Commelinaceae | *Commelina communis* F. Muell. | Clear heat and relieve toxicity, lower temperature | Whole plant | Dry |
| 73 | Commelinaceae | *Tradescantia spathacea* SW. | Clear heat and relieve toxicity | Flower | Dry |
| 74 | Cucurbitaceae | *Benincasa hispida* (Thunb.) Cogn. | Moisten intestines and relax bowls | Peel, seed | Dry |
| 75 | Cucurbitaceae | *Gynostemma pentaphyllum* (Thunb.) Makino | Anti-tumor, tonic | Aerial part | Dry |
| 76 | Cucurbitaceae | *Sechium edule* (Jacq.) Sw. | Soothe liver to regulate *qi* flow, harmonize stomach, resolve phlegm | Flower | Dry |
| 77 | Cucurbitaceae | *Siraitia grosvenorii* (Swingle) C. Jeffrey ex A.M. Lu & Zhi Y. Zhang | Clear lung and resolve phlegm, relieve sore throat and restore voice, moisten intestines and release bowls | Fruit | Dry |
| 78 | Cucurbitaceae | *Trichosanthes kirilowii* Maxim. | Resolve phlegm and relieve cough | Fruit, peel, seed | Dry |
| 79 | Cyperaceae | *Cyperus rotundus* Benth. | Soothe liver to regulate *qi*, regulate menstruation to relieve pain | Root | Dry |
| 80 | Cyperaceae | *Eleocharis dulcis* Hensch. | Clear heat and promote urination, cool blood and eliminate toxic materials, resolve phlegm and release bowls | Tuber | Fresh |
| 81 | Dioscoreaceae | *Dioscorea collettii* Hook. f. | Eliminate dampness and turbidity, expel wind-damp | Root | Dry |
| 82 | Dioscoreaceae | *Dioscorea fordii* Prain & Burkill | Nourish the spleen and stomach, promote production of fluid and nourish lung, tonify kidney and secure essence | Root | Dry |
| 83 | Dryopteridaceae | *Cyrtomium fortunei* J. Smith | Clear heat and relieve toxicity | Rhizome | Dry |
| 84 | Elaeagnaceae | *Hippophae rhamnoides* L. | Reduce blood pressure, promote digestion | Fruit | Dry or fresh |
| 85 | Equisetaceae | *Equisetum hyemale* L. | Clear liver and improve vision | Aerial part | Dry |
| 86 | Ericaceae | *Vaccinium fragile* Franch.,  *V. laetum* Diels,  *V. mandarinorum* Diels | Inoxidizabel | Fruit | Dry |
| 87 | Eucommiaceae | *Eucommia ulmoides* Oliv. | Tonify kidney and liver, strengthen tendons and bones, prevent abortion | Bark | Dry |
| 88 | Euphorbiaceae | *Phyllanthus emblica* L. | Anti-inflammation, digestion promoting | Fruit | Fresh or processed |
| 89 | Euphorbiaceae | *Sauropus spatulifolius* Beille | Soothe throat, stop cough | Leaf, flower | Dry |
| 90 | Fabaceae | *Abrus cantoniensis* Hance | Clear liver and promote urination | Whole plant | Dry |
| 91 | Fabaceae | *Canavalia gladiata* (Jacq.) DC. | Stop cough, tonic | Fruit | Dry |
| 92 | Fabaceae | *Gleditsia sinensis* Lam. | Relieve pathological heat and remove dampness through diruresis | Fruit | Dry |
| 93 | Fabaceae | *Glycine max* (L.) Merr. | Promote immunity, tonic, stop headache | Seed | Fermented |
| 94 | Fabaceae | *Glycyrrhiza uralensis* Fisch. ex DC. | Tonify spleen and replenish *qi*, dispel phlegm and arrest cough, relieve spasm and pain, clear heat and relieve toxicity, harmonize all medicinals | Root | Dry |
| 95 | Fabaceae | *Lablab purpureus* (L.) Sweet | Invigorate spleen and resolve dampness, harmonize the middle and dispel summer-heat | Fruit, flower | Dry |
| 96 | Fabaceae | *Phaseolus calcaratus* Roxb. | Promote diuresis to drain dampness and eliminate toxic materials, relieve swelling | Seed | Dry |
| 97 | Fabaceae | *Pueraria edulis* Pamp. | Relieve muscles and subdue fever, raise *yang* and stop diarrhea | Root | Dry |
| 98 | Fabaceae | *Senna tora* var. *obtusifolia* (L.) X.Y. Zhu | Clear heat and improve vision, moisten intestines and relax bowls | Seed | Dry |
| 99 | Fabaceae | *Sophora japonica* L. | Cool the blood and stop bleeding, clear away liver and large intestine meridians | Flower | Dry |
| 100 | Fabaceae | *Tadehagi triquetrum* (L.) H. Ohashi | Clear heat and promote diuresis to drain dampness, destroy parasites, clear away summer-heat | Aerial part | Dry |
| 101 | Fabaceae | *Vicia faba* L. | Relieve exterior and resolve dampness | Flower | Dry |
| 102 | Fabaceae | *Vigna radiata* (L.) R.Wilczek | Clear heat and relieve toxicity, eliminate summer-heat, induce dieresis, relieve agitation and stop thirst | Seed | Dry |
| 103 | Gentianaceae | *Gentiana scabra* Bunge | Clear heat and dry dampness, purge fire in liver and gallbladder | Root, rhizome | Dry |
| 104 | Geraniaceae | *Geranium wilfordii* Maxim. | Clear heat and relieve toxicity | Whole plant | Dry |
| 105 | Gingkoaceae | *Ginkgo biloba* L. | Astringe lung to relieve dyspnea, stop leukorrhagia, reduce unination | Leaf | Dry |
| 106 | Hamamelidaceae | *Liquidambar formosana* Hance | Regain consciousness | Fruit | Dry |
| 107 | Hypericaceae | *Hypericum japonicum* Thunb. | Anti-inflammatory and eliminate toxic materials | Whole plant | Dry |
| 108 | Iridaceae | *Belamcanda chinensis* (L.) Redouté | Clear heat and relieve toxicity, dispel phlegm and relieve sore throat | Rhizome | Dry |
| 109 | Juncaceae | *Juncus effusus* Schum. ex Schult. f. | Clear away liver-heat and lower the fire | Pith | Dry |
| 110 | Lamiaceae | *Agastache rugosa*  Kuntze | Relieve dampness, stimulate the appetite and relieve vomiting | Whole plant | Dry or fresh |
| 111 | Lamiaceae | *Elsholtzia ciliata* (Thunb.) Hyl. | disperse cold, relieve exterior，harmonize the middle and promote diuresis to drain dampness | Whole plant | Dry |
| 112 | Lamiaceae | *Lavandula angustifolia* Moench | Sedative, hypnotics | Whole plant | Dry |
| 113 | Lamiaceae | *Leonurus japonicus* Houtt. | Activate blood and dispel stasis, induce dieresis and alleviate edema | Whole plant | Dry |
| 114 | Lamiaceae | *Melissa officinalis* L. | Sedative, digestion promoting | Whole plant | Dry |
| 115 | Lamiaceae | *Mentha canadensis* L. | Eliminate toxic materials and relieve sore throat, dissipate mass and relieve swelling | Whole plant | Dry or fresh |
| 116 | Lamiaceae | *Mesona chinensis* Benth. | Clear heat and purge fire, promote urination and clear away summer-heat | Whole plant | Dry or fresh |
| 117 | Lamiaceae | *Perilla frutescens* L. ex B.D. Jacks. | Direct *qi* downward and resolve phlegm, relieve cough and dyspnea, moisten intestine and release bowls | Leaf | Dry or fresh |
| 118 | Lamiaceae | *Prunella vulgaris* Greene | Clear liver, purge fire, resolve knots, dissolve swelling, pacify liver and improve vision | Whole plant | Dry |
| 119 | Lamiaceae | *Salvia miltiorrhiza* Bunge | Promote blood circulation to remove blood stasis, cool the blood to relieve carbuncle, remove annoyance and tranquilize the mind | Root and rhizome | Dry |
| 120 | Lamiaceae | *Nepeta tenuifolia* Steud. | Expel wind-dampness | Whole plant | Dry |
| 121 | Lamiaceae | *Scutellaria baicalensis* Georgi | Clear heat and relieve toxicity | Whole plant | Dry |
| 122 | Laminariaceae | *Laminaria japonica* Areschoug | Anti-tumor, immunity promoting | Whole plant | Dry |
| 123 | Lardizabalaceae | *Akebia trifoliata*  (Thunb.) Koidz. | Induce dieresis, promote dieresis to relive stranguria, clear away heart-heat, promote menstruation and lactation | Stem | Dry or fresh |
| 124 | Liliaceae | *Aloe vera* var. *chinensis* (Haw.) Berg. | Purge and relax the bowels, clear liver and kill worms | Leaf | Fresh |
| 125 | Liliaceae | *Anemarrhena asphodeloides* Bunge | Strengthen stomach and lung | Bulb | Dry |
| 126 | Liliaceae | *Lilium brownii* var. *viridulum* Baker,  *L. lancifolium* Ker-Gawl. | Nourish yin and moisten lung, clear heart to induce tranquilization | Bulb | Dry |
| 127 | Liliaceae | *Ophiopogon japonicus* (L. f.) Ker-Gawl. | Smooth lung and nourish yin | Root | Dry |
| 128 | Liliaceae | *Polygonatum cyrtonema* Hua | Tonify *qi* and nourish yin, invigorate the spleen, moisten the lung, reinforce the kidney | Rhizome | Dry |
| 129 | Liliaceae | *Polygonatum odoratum* (Mill.) Druce | Nourish *yin* and moisten dryness, promote fluid production to relieve thirst | Rhizome | Dry |
| 130 | Lygodiaceae | *Lygodium japonicum* (Thunb.) Sw. | Clear heat and purge fire | Pollen | Dry |
| 131 | Magnoliaceae | *Houpoea officinalis* (Rehder & E.H. Wilson) N.H. Xia & C.Y. Wu | Clear heat and relieve toxicity | Flower | Dry |
| 132 | Magnoliaceae | *Magnolia liliiflora* Desr. | Regain consciousness | Flower | Dry |
| 133 | Moraceae | *Morus alba* L. | Dispel wind-heat, clear lung, moisten dryness, pacify liver and subdue *yang*, clear liver and improve vision. | Leaf, fruit | Dry or fresh |
| 134 | Moraceae | *Ficus carica* L. | Digestion promoting, anti-virus | Fruit | Dry |
| 135 | Moraceae | *Ficus microcarpa* L. f. | Expel wind-dampness and clear heat, cool blood and eliminate toxic materials | Aerial root | Dry |
| 136 | Myrtaceae | *Cleistocalyx operculatus* (Roxb.) Merr. & L.M. Perry | Clear heat and relieve toxicity, stimulate appetite | Flower, branch | Dry |
| 137 | Myrtaceae | *Syzygium jambos* (L.) Alston | Warm stomach and invigorate spleen, stop diarrhea, warm lung and stop cough, relieve swelling | Bark, peel, seed | Dry or fresh |
| 138 | Nymphaeaceae | *Nelumbo nucifera* Gaertn. | Clear summer-heat and promote diuresis to drain dampness, invigorate spleen and replenish *qi*, resolve blood-stasis and stop bleeding, lose weight | Leaf, fruit, seed | Dry or fresh |
| 139 | Oleaceae | *Forsythia suspensa* Vahl. | Clear heat and relieve toxicity, cure swelling and dissipate nodulation | Fruit | Dry |
| 140 | Oleaceae | *Ligustrum lucidum* Hort. ex K.Koch | Nourish liver and kidney, improve vision and blacken hair | Fruit | Dry |
| 141 | Oleaceae | *Osmanthus fragrans* Lour. | Activate blood and resolve stasis | Flower | Dry |
| 142 | Orchidaceae | *Dendrobium officinale* Kimura & Migo,  *D. chrysanthum* Wall.,  *D. fimbriatum* Dalzell,  *D. loddigesii* Rolfe,  *D. nobile* Lindl*.* | Strengthen stomach and promote fluid production, nourish *yin* and clear heat | Stem | Dry and processed |
| 143 | Orchidaceae | *Nervilia fordii* Schltr. | Clear heat | Whole plant | Dry |
| 144 | Paeoniaceae | *Paeonia lactiflora* Pall.,  *P. veitchii* Lynch | Clear heat, cool blood, resolve stasis and stop pain | Root | Removed surface and dry |
| 145 | Paeoniaceae | *Paeonia sterniana* H.R. Fletcher | Nourish blood and regulate menstruation, suppress liver to relieve pain, astringe *yin* to arrest sweating | Root | Removed surface and dry |
| 146 | Paeoniaceae | *Paeonia suffruticosa* Andrews | Clear heat, cool blood, activate blood and resolve stasis | Root bark | Removed surface and dry |
| 147 | Palmae | *Areca catechu* L. | Replenish *qi* and moisten intestines | Peel | Dry |
| 148 | Pandanaceae | *Pandanus tectorius* Sol. ex Balf. f. | Nourish kidney | Fruit | Dry or fresh |
| 149 | Pedaliaceae | *Sesamum indicum* L. | Tonic | Seed | Dry |
| 150 | Plantaginaceae | *Plantago asiatica* Turcz. | Clear heat and dampness, induce diuresis | Whole plant, seed | Dry or fresh |
| 151 | Poaceae | *Bambusa tuldoides* Munro,  *Dendrocalamus beecheyana* var. *pubescens* (P.P. Li) Keng f.,  *Phyllostachys glauca* McClure | Clear heat and resolve phlegm, alleviate restlessness and relieve vomiting | Stem | Dry |
| 152 | Poaceae | *Coix lacryma-jobi* L. | Promote dieresis to drain dampness, invigorate the spleen to relieve diarrhea, treat Bi-syndrome, clear away heat to drain the pus | Kernel | Dry |
| 153 | Poaceae | *Imperata cylindrica* (L.) P. Beauv. | Clear heat and promote urination | Rhizome | Dry or fresh |
| 154 | Poaceae | *Lophatherum gracile* Brongn. | Clear heat, sedative | Leaf, root | Dry or fresh |
| 155 | Poaceae | *Oryza sativa* Hochst. ex Steud. | Nourish stomach | Seed | Dry |
| 156 | Poaceae | *Oryza sativa* var. *glutinosa* Matsum. | Arrest sweating | Root | Dry |
| 157 | Poaceae | *Phragmites australis* (Cav.) Steud. | Clear away summer-heat, anti-inflammatory | Rhizome | Dry |
| 158 | Poaceae | *Pogonatherum crinitum* Kunth | Clear heat and promote urination | Whole plant | Dry |
| 159 | Poaceae | *Saccharum sinense* Roxb*.* | Clear heat and purge fire, expel dampness and eliminate toxic materials | Stem, young leaf | Dry |
| 160 | Poaceae | *Triticum aestivum* L. | Promote digestion, harmonize the middle energizer, stop lactation and disperse swelling | bud | Dry |
| 161 | Poaceae | *Zea mays* L. | Expel dampness and induce diuresis | Stigma | Dry |
| 162 | Polygonaceae | *Fagopyrum dibotrys* (D.Don) Hara | Clear heat and relieve toxicity | Rhizome | Dry |
| 163 | Polygonaceae | *Polygonum aviculare* L. | Anti-bacteria, clear heat | Whole plant | Dry |
| 164 | Polygonaceae | *Polygonum chinense* L. | Clear heat and purge fire | Whole plant | Dry |
| 165 | Polygonaceae | *Polygonum cuspidatum* Willd. ex Soler. | Drain the gallbladder to treat jaundice, clear away heat and eliminate toxic materials, activate blood and resolve stasis, resolve phlegm to stop cough | Root, rhizome | Dry |
| 166 | Polygonaceae | *Polygonum hydropiper* L. | Clear heat and promote diuresis to drain dampness | Whole plant | Dry or fresh |
| 167 | Polygonaceae | *Fallopia multiflora* (Thunb.) Haraldson | Moisten intestines and relax bowls | Root | Dry |
| 168 | Polygonaceae | *Rheum palmatum* L. | Remove stagnation by purgation, clear heat and discharge fire, cool blood and remove toxin, active blood and remove blood stasis, clear and purge damp-heat | Root | Dry |
| 169 | Polypodiaceae | *Pyrrosia lingua* Farw. | Clear heat and promote urination, promote diuresis to relieve stranguria | Whole plant | Dry |
| 170 | Polyporaceae | *Wolfiporia extensa* (Peck) Ginns | Promote dieresis to drain dampness, invigorate the spleen and sedate the mind | Fruit body | Dry |
| 171 | Portulacaceae | *Portulaca oleracea* L. | Clear heat and relieve toxicity, cool blood and stop dysentery | Whole plant | Dry |
| 172 | Primulaceae | *Lysimachia christiniae* Hance | Promote urination, eliminate dampness to treat jaundice, eliminate toxic materials and relieve swelling | Whole plant | Dry |
| 173 | Pteridaceae | *Doryopteris ludens* (Wall. ex Hook.) J. Sm. | Clear heat and promote diuresis to drain dampness | Whole plant | Dry |
| 174 | Punicaceae | *Punica granatum* L. | Promote digestion, stop blooding | Peel | Dry or fresh |
| 175 | Ranunculaceae | *Pulsatilla chinensis* (Bunge) Regel | Clear heat, relieve toxicity, cool blood and stop dysentery | Root | Dry |
| 176 | Ranunculaceae | *Cimicifuga foetida* L. | Disperse wind andrelease superficies | Rhizome | Dry |
| 177 | Ranunculaceae | *Coptis chinensis* Franch. | Clear heat, dry dampness, purge fire and relieve toxicity | Root and rhizome | Dry |
| 178 | Rhamnaceae | *Berchemia lineata* DC. | Stop cough | Stem, root | Dry |
| 179 | Rhamnaceae | *Ziziphus jujuba* Mill. | Tonify the middle and replenish *qi*, nourish blood and induce tranquilization | Fruit | Dry |
| 180 | Rosaceae | *Agrimonia pilosa* Ledeb. | Stop bleeding by astringing, relieve dysentery and kill trichomonad | Whole plant | Dry |
| 181 | Rosaceae | *Chaenomeles sinensis* (Thouin) Koehne,  *C. speciosa* (Sweet) Nakai | Soothe tendons and activate collaterals, harmonize stomach and resolve dampness | Fruit | Dry or fresh |
| 182 | Rosaceae | *Crataegus pinnatifida* Bunge | Promote digestion and relieve dyspepsia, invigorate blood and disperse blood-stasis | Fruit | Dry |
| 183 | Rosaceae | *Eriobotrya japonica* (Thunb.) Lindl. | Clear lung and relieve cough, check adverse rise of *qi* to stop vomiting | Leaf | Dry or fresh |
| 184 | Rosaceae | *Armeniaca vulgaris* Lam*.* | Relieve cough and dyspnea, moisten intestine and release bowels | Kernel | Dry |
| 185 | Rosaceae | *Prunus mume* Siebold & Zucc. | Astringe the lung, astringe the intestine, promote the production of body fluid, relieve ascaris colic | Fruit | Processed |
| 186 | Rosaceae | *Prunus sibirica* L. | Warm lung and disperse cold | Kernel | Dry |
| 187 | Rosaceae | *Rosa laevigata* Michx. | Clear heat and purge fire | Fruit, root | Dry |
| 188 | Rosaceae | *Rosa rugosa* Thunb. | Soothe liver to remove depression, promote blood circulation and stop pain | Flower, fruit | Dry |
| 189 | Rubiaceae | *Gardenia jasminoides* Retz. | Clear heat and relieve exterior, promote eruption | Fruit, flower | Dry |
| 190 | Rubiaceae | *Hedyotis diffusa* Willd. | Clear heat and relieve toxicity, excrete damp and relieve stranguria | Whole plant | Dry |
| 191 | Rubiaceae | *Paederia scandens* (Lour.) Merr. | Clear liver and clear away summer-heat | Whole plant | Dry or fresh |
| 192 | Rubiaceae | *Uncaria sinensis* Havil.,  *U. macrophylla* Wall.,  *U. rhynchophylla* Miq. | Disperse wind and discharge heat, tranquilize | Stem node with thorns | Dry |
| 193 | Rutaceae | *Citrus aurantium* L. | Break *qi*, remove food retention, resolve phlegm and dissipate mass | Fruit | Dry |
| 194 | Rutaceae | *Citrus maxima* (Burm.) Merr. | Stop cough | Fruit | Dry |
| 195 | Rutaceae | *Citrus medica* L. | Stop cough, anti-asthma | Fruit | Dry |
| 196 | Rutaceae | *Citrus reticulata* Blanco | Stop cough, strengthen stomach | Green and red peel | Dry |
| 197 | Rutaceae | *Phellodendron amurense* Rupr. | Anti-bacteria, anti-virus | Bark | Dry |
| 198 | Rutaceae | *Ruta graveolens* L. | Clear heat and expel wind-dampness, activate blood and promote urination, relieve swelling and toxicity | Whole plant | Dry |
| 199 | Sabiaceae | *Sabia japonica* Maxim. | Clear heat and relieve toxicity | Stem | Dry |
| 200 | Saururaceae | *Houttuynia cordata* Thunb. | Clear heat and relieve toxicity | Whole plant | Dry or fresh |
| 201 | Schisandraceae | *Schisandra chinensis* (Turcz.) Baill. | Astringe and strengthen, benefit *qi* and promote the production of body fluid, tonify the kidney and calm the mind | Fruit | Dry |
| 202 | Scrophulariaceae | *Rehmannia glutinosa* Steud. | Clear heat and relieve toxicity | Root | Dry |
| 203 | Scrophulariaceae | *Scrophularia ningpoensis* Hemsl. | Cool blood, nourish *yin*, purge fire, and relieve toxicity | Root | Dry |
| 204 | Scrophurariaceae | *Striga asiatica* (L.) Kuntze | Pacify liver and clear heat, remove food retention | Whole plant | Dry |
| 205 | Selaginellaceae | *Selaginella tamariscina* (P. Beauv.) Spring | Activate blood and resolve stasis | Whole plant | Dry |
| 206 | Smilaceae | *Smilax china* L. | Alleviate consumptive thirst | Root | Dry |
| 207 | Smilaceae | *Smilax glabra* Roxb. | Clear heat and relieve toxicity, ease joint movement | Root | Dry |
| 208 | Solanaceae | *Lycium chinense* Mill. | Cool blood, relieve bone-steaming, clear lung fire | Root bark | Dry |
| 209 | Stemonaceae | *Stemona tuberosa* Lour. | Moisten lung and relieve cough, kill lice and worms | Root | Dry |
| 210 | Sterculiaceae | *Helicteres angustifolia* L. | Clear heat and purge fire | Root or aerial part | Dry |
| 211 | Sterculiaceae | *Sterculia lychnophora* Hance | Clear lung and resolve phlegm, relieve sore throat and restore voice, moisten intestines and release bowls | Fruit | Dry |
| 212 | Tiliaceae | *Microcos stauntoniana* G.Don | Removing pathogenic heat to promote digestion | Leaf | Dry |
| 213 | Tremellaceae | *Tremella fuciformis* Berk. | Alleviate consumptive thirst, stop cough | Fruit body | Dry |
| 214 | Trilliaceae | *Paris polyphylla* var. *yunnanensis* (Franch.) Hand.-Mazz. | Anti-virus, stopping blood, treating traumatic injury | Rhizome | Dry |
| 215 | Valerianaceae | *Patrinia scabiosifolia* Fisch. ex Trevir. | Clear heat and relieve toxicity | Whole plant | Dry |
| 216 | Verbenaceae | *Clerodendrum chinense* (Osbeck) Mabb. | Expel wind-dampness and activate blood, relieve swelling and hypertension | Root, leaf | Dry |
| 217 | Verbenaceae | *Vitex negundo* L. | Clear heat and purge fire | Leaf | Dry |
| 218 | Zingiberaceae | *Alpinia officinarum* Hance | Warming the middle energizer and relieve pain | Rhizome | Dry |
| 219 | Zingiberaceae | *Amomum testaceum* Ridl*.* | Promote *qi* flow and resolve dampness, stop vomitting | Kernel | Dry |
| 220 | Zingiberaceae | *Amomum villosum* Lour. | Resolve dampness, move *qi*, warm the middle, check diarrhea, prevent abortion | Fruit | Dry |
| 221 | Zingiberaceae | *Curcuma aromatica* Salisb. | Activate blood and relieve pain, promote *qi* and disperse the stagnated *qi*, clear heart and cool blood, excrete bile and disperse jaundice | Rhizome | Dry |
| 222 | Zingiberaceae | *Zingiber officinalis* Roscoe | Dispel cold, release superficies, warm the middle, arrest vomiting, resolve phlegm and stop cough | Rhizome | Dry or fresh |
