# Supplementary material for: Ethnobotanical survey of cooling herbal drinks from southern China
Source: J Ethnobiol Ethnomed. 2013 Dec 19;9:82. doi: 10.1186/1746-4269-9-82 (PMC3926322; doi:10.1186/1746-4269-9-82)
Supplement: Additional file 2: Table S2 — Habitat information and conservation need of plants used for cooling herbal drinks in Lingnan region of southern China. [file 1746-4269-9-82-S2.doc]

**Additional file 2: Table S2. Habitat information and conservation need of plants used for cooling herbal drinks in Lingnan region of southern China**

(Numbers corresponded to those in Table 1)

| **No.** | **Scientific name** | **Chinese name** | **Wild / cultivated** | **Habitat** | **Conservation need** |
| --- | --- | --- | --- | --- | --- |
| **Acanthaceae** | | | | | |
| 1 | *Strobilanthes cusia* | Ban Lan Gen 板蓝根 | Wild and cultivated | Usually in moist wooded places |  |
| 2 | *Dicliptera chinensis* | Gou Gan Cai 狗肝菜 | Wild | Streamsides, trailsides |  |
| 3 | *Rungia pectinata* | Hai Er Cao 孩儿草 | Wild | Wastelands |  |
| **Acoraceae** | | | | | |
| 4 | *Acorus tatarinowii* | Shi Chang Pu石菖蒲 | Wild | River banks, wetlands, wet cliffs |  |
| **Alismataceae** | | | | | |
| 5 | *Alisma orientale* | Ze Xie 泽泻 | Wild | Margins of lakes, ponds, marshes, channels |  |
| **Alliaceae** | | | | | |
| 6 | *Allium macrostemon* | Jiu Bai 薤白 | Wild and cultivated | Meadows, stream banks, damp places |  |
| 7 | *Allium sativum* | Suan蒜 | Cultivated | Vegetable gardens, homegardens |  |
| **Amaranthaceae** | | | | | |
| 8 | *Achyranthes aspera* | Tu Niu Xi 土牛膝 | Wild | Hillsides, waste places, roadsides, riverbanks |  |
| 9 | *Achyranthes bidentata* | Niu Xi牛膝 | Wild | Hillsides |  |
| 10 | *Amaranthus spinosus* | Le Xian Cai 簕苋菜 | Wild | Waste places, gardens |  |
| 11 | *Celosia cristata* | Ji Guan Hua鸡冠花 | Cultivated | Vegetable gardens, homegardens |  |
| **Apiaceae** | | | | | |
| 12 | *Angelica biserrata* | Du Huo独活 | Wild and cultivated | Sparse shrubby thickets, damp slopes |  |
| 13 | *Angelica dahurica* | Bai Zhi白芷 | Wild and cultivated | Forest margins, valley grasslands, streamsides |  |
| 14 | *Angelica sinensis* | Dang Gui当归 | Wild and cultivated | forests, shrubby thickets |  |
| 15 | *Bupleurum chinense* | Chai Hu柴胡 | Wild | Grasslands, stream banks, sunny slopes, roadsides |  |
| 16 | *Centella asiatica* | Ji Xue Cao积雪草 | Wild | Shady, wet, grassy places, river margins |  |
| 17 | *Daucus carota* subsp. *sativus* | Hu Luo Bo胡萝卜 | Cultivated | Dry fields, vegetable gardens, homegardens |  |
| 18 | *Foeniculum vulgare* | Hui Xiang茴香 | Cultivated | Vegetable gardens, homegardens |  |
| 19 | *Ligusticum sinense* | Chuan Xiong川芎 | Wild and cultivated | Forests, montane scrub, grassy slopes, streamsides, moist roadsides |  |
| 20 | *Notopterygium incisum* | Jiang Huo羌活 | Wild | Forest margins, scrub | VU |
| 21 | *Peucedanum decursivum* | Qian Hu前胡 | Wild | Forest margins, shrubby thickets, slopes, streamsides |  |
| 22 | *Saposhnikovia divaricata* | Fang Feng防风 | Wild | Scrub, hillsides, grasslands, stony slopes |  |
| **Apocynaceae** | | | | | |
| 23 | *Plumeria rubra* | Ji Dan Hua鸡蛋花 | Cultivated | Roadsides, homegardens |  |
| 24 | *Trachelospermum jasminoides* | Luo Shi Teng络石藤 | Wild | Sunny edges of forests, brushwoods |  |
| **Aquifoliaceae** | | | | | |
| 25 | *Ilex asprella* | Gang Mei Gen岗梅根 | Wild | Sparse forests on slopes, shrubby areas, roadsides |  |
| 26 | *Ilex kaushue* | Ku Ding Cha苦丁茶 | Wild | Dense forests |  |
| 27 | *Ilex pubescens* | Mao Dong Qing毛冬青 | Wild | Evergreen broad-leaved forests, forest margins, shrubby areas, streamsides, roadsides |  |
| 28 | *Ilex rotunda* | Jiu Bi Ying救必应 | Wild | Evergreen broad-leaved forests, forest margins on mountain slopes |  |
| **Araceae** | | | | | |
| 29 | *Pinellia ternata* | Jiang Ban Xia姜半夏 | Wild and cultivated | Grasslands, secondary forests, wastelands, cultivated lands |  |
| **Araliaceae** | | | | | |
| 30 | *Panax notoginseng* | San Qi三七 | Cultivated | Mountain slopes with artificial shelters |  |
| 31 | *Panax quinquefolius* | Xi Yang Shen西洋参 | Cultivated | Mountain slopes with artificial shelters |  |
| 32 | *Parthenocissus tricuspidata* | Pa Shan Hu爬山虎 | Wild and cultivated | Shrublands, cliffs, rocky hillsides |  |
| 33 | *Schefflera bodinieri* | Ya Jiao Mu鸭脚木 | Wild | Dense forests on slopes or in valleys |  |
| 34 | *Tetrapanax papyrifer* | Tong Cao通草 | Wild | Mixed thickets |  |
| **Asparagaceae** | | | | | |
| 35 | *Asparagus cochinchinensis* | Tian Men Dong天门冬 | Wild | Thinly forested slopes, roadsides, waste fields |  |
| **Asteraceae** | | | | | |
| 36 | *Arctium lappa* | Niu Bang牛蒡 | Wild and cultivated | Near villages, roadsides, near rivers, wet and waste places, forest margins, thickets, valleys, slopes |  |
| 37 | *Artemisia annua* | Qing Hao青蒿 | Wild | Hills, waysides, wastelands, outer forest margins, steppes, forest steppes, dry floodlands, terraces, semidesert steppes, rocky slopes, roadsides, saline soils |  |
| 38 | *Artemisia argyi* | Ai Ye艾叶 | Wild | Waste places, roadsides, slopes, hills, steppes, forest steppes |  |
| 39 | *Artemisia scoparia* | Yin Chen茵陈 | Wild | Slopes, forest margins, roadsides, steppes, terraces, desert margins, dry riverbeds |  |
| 40 | *Atractylodes lancea* | Cang Shu苍术 | Wild and cultivated | Grasslands, forests, thickets, rock crevices |  |
| 41 | *Atractylodes macrocephala* | Bai Shu白术 | Wild and cultivated | Grasslands, forests | VU |
| 42 | *Carthamus tinctorius* | Hong Hua红花 | Wild and cultivated | Widely cultivated in China and occasionally naturalized in saline and alkaline soils in dry and cold conditions |  |
| 43 | *Chrysanthemum indicum* | Ye Ju Hua野菊花 | Wild | Grasslands on mountain slopes, thickets, wet places by rivers, fields, roadsides, saline places by seashores, under shrubs |  |
|  | *Chrysanthemum lavandulaefolium* |  |  |  |  |
| 44 | *Chrysanthemum morifolium* | Ju Hua菊花 | Wild and cultivated | Farming fields; roadsides, mountain slopes |  |
| 45 | *Cirsium japonicum* | Da Ji大蓟 | Wild | Forests, forest margins, thickets, grasslands, wastelands, farmlands, roadsides, streamsides |  |
| 46 | *Cirsium arvense* var. *integrifolium* | Xiao Ji小蓟 | Wild | Mountain slopes, by rivers, wet lands, farmlands |  |
| 47 | *Eupatorium fortunei* | Pei Lan佩兰 | Wild and cultivated | Thickets or roadside ditches |  |
| 48 | *Elephantopus scaber* | Di Dan Cao地胆草 | Wild | Often on open slopes, roadsides or forest margins in valleys |  |
| 49 | *Emilia sonchifolia* | Yi Dian Hong一点红 | Wild | Weedy slopes, roadsides, field margins, sandy places |  |
| 50 | *Pseudognaphalium affine* | Fo Er Cao佛耳草 | Wild | A weed of wastelands and cultivated fields |  |
| 51 | *Spilanthes acmella* | Jin Niu Kou金纽扣 | Wild | Fields, wastelands, roadsides, forest margins |  |
| 52 | *Siegesbeckia orientalis* | Xi Qian豨签 | Wild | Fields, thickets, forest margins, forests |  |
| 53 | *Taraxacum officinale* | Pu Gong Ying蒲公英 | Wild | Roadsides, mountain slopes |  |
| 54 | *Tussilago farfara* | Kuan Dong hua款冬花 | Wild | Wet places, forest understories, valleys |  |
| 55 | *Xanthium strumarium* | Cang Er苍耳 | Wild | Damp or seasonally wet often alkaline soils, wastelands, margins of agriculture |  |
| **Berberidaceae** | | | | | |
| 56 | *Mahonia fortunei* | Shi Da Gong Lao十大功劳 | Cultivated | Dry fields, mountain slopes |  |
| **Bignoniaceae** | | | | | |
| 57 | *Oroxylum indicum* | Mu Hu Die木蝴蝶 | Wild | Tropical and subtropical low altitude open forests, roadsides, slopes |  |
| **Bombacaceae** | | | | | |
| 58 | *Bombax ceiba* | Mu Mian Hua木棉花 | Wild and cultivated | Hot dry river valleys, savanna |  |
| **Boraginaceae** | | | | | |
| 59 | *Lithospermum erythrorhizon* | Zi Cao紫草 | Wild | Meadows on slopes |  |
| **Brassicaceae** | | | | | |
| 60 | *Brassica juncea* | Jie Cai芥菜 | Wild and cultivated | Fields, waste places, roadsides |  |
| 61 | *Isatis tinctoria* | Da Qing Ye大青叶 | Wild | Fields, pastures, roadsides, waste places |  |
| 62 | *Raphanus sativus* | Lai Fu Zi莱菔子 | Cultivated | Fields, roadsides, waste areas |  |
| **Burseraceae** | | | | | |
| 63 | *Canarium album* | Qing Guo青果 | Wild and cultivated | Forests on slopes, valleys |  |
| **Campanulaceae** | | | | | |
| 64 | *Adenophora stricta* | Sha Shen沙参 | Wild | Meadows, forest margins, scrub, open mountain slopes, forests, among grasses, in rock crevices |  |
| 65 | *Codonopsis pilosula* | Dang Shen党参 | Cultivated | Forests, thickets, meadows or scrub at forest margins |  |
| 66 | *Platycodon grandiflorus* | Jie Geng桔梗 | Cultivated | Sunny herb communities, thickets, rarely in forests |  |
| **Cannabiaceae** | | | | | |
| 67 | *Cannabis sativa* | Da Ma大麻 | Cultivated | Uplands |  |
| **Caprifoliaceae** | | | | | |
| 68 | *Lonicera confusa*, | Jin Yin Hua金银花 | Wild and cultivated | Mountain slopes, mixed forests, scrub, roadsides of plains, riversides |  |
|  | *L. hypoglauca*, |  |  |  |  |
|  | *L. japonica* |  |  |  |  |
| **Caryophyllaceae** | | | | | |
| 69 | *Pseudostellaria heterophylla* | Tai Zi Shen太子参 | Wild and cultivated | Mountain valleys, moist shaded forests |  |
| 70 | *Vaccaria hispanica* | Wang Bu Liu Xing王不留行 | Wild | Wheat fields |  |
| **Combretaceae** | | | | | |
| 71 | *Quisqualis indica* | Shi Jun Zi使君子 | Wild and cultivated | Rain forests, low woods, thickets, hedges, mountains, dry hillsides, riversides, roadsides, wasteland |  |
| **Commelinaceae** | | | | | |
| 72 | *Commelina communis* | Ya Zhi Cao鸭跖草 | Wild | Humid places |  |
| 73 | *Tradescantia spathacea* | Bang Lan Hua蚌兰花 | Naturalized and cultivated | Roadsides, mountain slopes |  |
| **Cucurbitaceae** | | | | | |
| 74 | *Benincasa hispida* | Dong Gua冬瓜 | Cultivated | Dry farming fields, vegetable gardens, homegardens |  |
| 75 | *Gynostemma pentaphyllum* | Jiao Gu Lan绞股蓝 | Wild | Forests, thickets or roadsides on mountain slopes |  |
| 76 | *Sechium edule* | Fo Shou佛手 | Cultivated | Vegetable gardens, homegardens |  |
| 77 | *Siraitia grosvenorii* | Luo Han Guo罗汉果 | Cultivated | Forests on mountain slopes, riversides, thickets |  |
| 78 | *Trichosanthes kirilowii* | Gua Lou栝楼 | Wild | Open forests, shrublands, grasslands and fields beside villages |  |
| **Cyperaceae** | | | | | |
| 79 | *Cyperus rotundus* | Xiang Fu Zi香附子 | Wild | Grasslands, wet or dry areas on mountain slopes, stream margins, along trails, sandbanks, ditch margins, water margins in valleys, paddy field margins |  |
| 80 | *Eleocharis dulcis* | Bi Qi荸荠 | Wild | Field margins, lake margins, commonly cultivated |  |
| **Dioscoreaceae** | | | | | |
| 81 | *Dioscorea collettii* | Cha Rui Shu Yu叉蕊薯蓣 | Wild | Mixed forests, secondary Quercus forests, scrub forests, mountain slopes |  |
| 82 | *Dioscorea fordii* | Huai Shan淮山 | Wild and cultivated | Mixed forests, mountain slopes, valleys, along rivers, roadsides |  |
| **Dryopteridaceae** | | | | | |
| 83 | *Cyrtomium fortunei* | Guan Zhong贯众 | Wild | Hillside, forests, ditch stream |  |
| **Elaeagnaceae** | | | | | |
| 84 | *Hippophae rhamnoides* | Sha Ji沙棘 | Wild | River banks and terraces, dry river beds, forest margins, thickets on mountain slopes, moraines, meadows at highest elevations |  |
| **Equisetaceae** | | | | | |
| 85 | *Equisetum hyemale* | Mu Zei木贼 | Wild | Slope forests, wet places, stream, foul land |  |
| **Ericaceae** | | | | | |
| 86 | *Vaccinium fragile*, | Yue Ju越橘 | Wild | Pinus and Quercus forests, thickets, open grassy slopes |  |
|  | *V. laetum*, |  |  |  |  |
|  | *V. mandarinorum* |  |  |  |  |
| **Eucommiaceae** | | | | | |
| 87 | *Eucommia ulmoides* | Du Zhong杜仲 | widely cultivated | Mixed forests, sparse forests, thickets, lower mountains, ridges, valleys, dry ravines, fields |  |
| **Euphorbiaceae** | | | | | |
| 88 | *Phyllanthus emblica* | Yu Gan Zi余甘子 | Wild | Dry open sparse forests or scrub, village groves |  |
| 89 | *Sauropus spatulifolius* | Long Li Ye龙利叶 | Cultivated | Vegetable gardens, homegardens |  |
| **Fabaceae** | | | | | |
| 90 | *Abrus cantoniensis* | Ji Gu Cao鸡骨草 | Wild | Mountain, shrub |  |
| 91 | *Canavalia gladiata* | Dao Dou刀豆 | Cultivated | Homegardens |  |
| 92 | *Gleditsia sinensis* | Zao Jiao皂角 | Wild | Mountain slopes, forests, valleys, near roads |  |
| 93 | *Glycine max* | Dan Dou Chi淡豆豉 | Cultivated | Dry fields, farming lands |  |
| 94 | *Glycyrrhiza uralensis* | Gan Cao甘草 | Wild | Sandy lands, dry riverbanks, grasslands on hills | Regional Red Data Book |
| 95 | *Lablab purpureus* | Bai Bian Dou白扁豆 | Cultivated | Homegardens, vegetable gardens |  |
| 96 | *Phaseolus calcaratus* | Chi Xiao Dou赤小豆 | Cultivated | Farming lands |  |
| 97 | *Pueraria edulis* | Ge Gen葛根 | Wild | Forests |  |
| 98 | *Senna tora* var*. obtusifolia* | Jue Ming Zi决明子 | Wild | Mountain slopes, wastelands, riverbank sand |  |
| 99 | *Sophora japonica* | Huai Hua槐花 | Cultivated | Roadsides |  |
| 100 | *Tadehagi triquetrum* | Hu Lu Cha葫芦茶 | Wild | Wastelands, forest margins, roadsides |  |
| 101 | *Vicia faba* | Nan Dou Hua南豆花 | Cultivated | Dry fields, farming lands; vegetable gardens |  |
| 102 | *Vigna radiata* | Lǜ Dou绿豆 | Wild and cultivated | Open wastelands, roadsides, thicket margins |  |
| **Gentianaceae** | | | | | |
| 103 | *Gentiana scabra* | Long Dan Cao龙胆草 | Wild | River banks, grassland and roadside slopes, moist meadows, scrub, forest margins, forests |  |
| **Geraniaceae** | | | | | |
| 104 | *Geranium wilfordii* | Lao Guan Cao老鹳草 | Wild | Scrub, meadows, by water, weedy areas |  |
| **Gingkoaceae** | | | | | |
| 105 | *Ginkgo biloba* | Yin Xing银杏 | Cultivated | Scattered in broad-leaved forests and valleys on acidic, well-drained, yellow loess (pH = 5-5.5) |  |
| **Hamamelidaceae** | | | | | |
| 106 | *Liquidambar formosana* | Lu Lu Tong路路通 | Wild and cultivated | Sunny places, near villages, montane forests |  |
| **Hypericaceae** | | | | | |
| 107 | *Hypericum japonicum* | Tian Ji Huang田基黄 | Wild | Rice fields, ditches, marshes, grasslands, waste places |  |
| **Iridaceae** | | | | | |
| 108 | *Belamcanda chinensis* | She Gan射干 | Cultivated | Farming fields, homegardens |  |
| **Juncaceae** | | | | | |
| 109 | *Juncus effusus* | Deng Xin Cao灯芯草 | Wild | Forest margins, wet grasslands, pools, morasses, lake margins, river banks, fields, rice fields |  |
| **Lamiaceae** | | | | | |
| 110 | *Agastache rugosa* | Huo Xiang藿香 | Cultivated | Farming fields, homegardens |  |
| 111 | *Elsholtzia ciliata* | Xiang Ru香薷 | Wild | Hills, waste areas, sunny terraces, riverbanks, forests |  |
| 112 | *Lavandula angustifolia* | Xun Yi Cao薰衣草 | Cultivated | Farming fields, homegardens |  |
| 113 | *Leonurus japonicus* | Yi Mu Cao益母草 | Wild | Sunny areas |  |
| 114 | *Melissa officinalis* | Xiang Feng Ye香蜂叶 | Cultivated | Farming fields, homegardens |  |
| 115 | *Mentha canadensis* | Bo He薄荷 | Wild and cultivated | Wet areas |  |
| 116 | *Mesona chinensis* | Xian Cao仙草 | Wild | Ravines, grassy, dry, and sandy areas |  |
| 117 | *Perilla frutescens* | Zi Su紫苏 | Cultivated | Waste areas |  |
| 118 | *Prunella vulgaris* | Xia Ku Cao夏枯草 | Wild | Open slopes, grasslands, wet streamsides, forest margins, thickets |  |
| 119 | *Salvia miltiorrhiza* | Dan Shen丹参 | Wild | Hillsides, streamsides, forests |  |
| 120 | *Nepeta tenuifolia* | Jing Jie荆芥 | Wild | Sloping forest margins, valleys |  |
| 121 | *Scutellaria baicalensis* | Huang Qin黄芩 | Wild | Sunny grassy slopes, waste and cultivated areas |  |
| **Laminariaceae** | | | | | |
| 122 | *Laminaria japonica* | Kun Bu昆布 | Wild and cultivated | water |  |
| **Lardizabalaceae** | | | | | |
| 123 | *Akebia trifoliata* | San Ye Mu Tong三叶木通 | Wild | Semideciduous forest margins, open forest along valleys, scrub on hillsides, by streams |  |
| **Liliaceae** | | | | | |
| 124 | *Aloe vera* var. *chinensis* | Lu Hui芦荟 | Wild and cultivated | Lowland fields, homegardens, indoors |  |
| 125 | *Anemarrhena asphodeloides* | Zhi Mu知母 | Wild and cultivated | Scrub, grassy slopes, steppes, sunny and sandy hillsides |  |
| 126 | *Lilium brownii* var. *viridulum*, | Bai He百合 | Cultivated | Sparse forests, grassy slopes, hillsides along ravines, wastelands around villages |  |
|  | *L. lancifolium* |  |  |  |  |
| 127 | *Ophiopogon japonicus* | Mai Dong麦冬 | Wild and cultivated | Forests, dense scrub in ravines, moist and shady places on slopes and along streams, cliffs |  |
| 128 | *Polygonatum cyrtonema* | Duo Hua Huang Jing多花黄精 | Wild | Forests, thickets, shaded slopes |  |
| 129 | *Polygonatum odoratum* | Yu Zhu玉竹 | Wild and cultivated | Forests, shaded slopes |  |
| **Lygodiaceae** | | | | | |
| 130 | *Lygodium japonicum* | Jin Sha Teng金沙滕 | Wild | Forests, roadsides, mountain slopes |  |
| **Magnoliaceae** | | | | | |
| 131 | *Houpoea officinalis* | Hou Pu厚朴 | Cultivated | Dry fields, mountain slopes |  |
| 132 | *Magnolia liliiflora* | Xin Yi Hua辛夷花 | Wild and cultivated | Forest margins, slopes | VU |
| **Moraceae** | | | | | |
| 133 | *Morus alba* | Sang桑 | Cultivated | Irrigated fields, farming lands |  |
| 134 | *Ficus carica* | Wu Hua Guo无花果 | Cultivated | Dry fields, homegardens |  |
| 135 | *Ficus microcarpa* | Rong Shu Xu榕树须 | Wild and cultivated | Mountains, plains |  |
| **Myrtaceae** | | | | | |
| 136 | *Cleistocalyx operculatus* | Shui Weng Hua水翁花 | Wild and cultivated | Forests, streams |  |
| 137 | *Syzygium jambos* | Pu Tao蒲桃 | Wild and cultivated | Mixed forests, mountain slopes, riversides, river valleys |  |
| **Nymphaeaceae** | | | | | |
| 138 | *Nelumbo nucifera* | Lian莲 | Cultivated | Lakes, ponds |  |
| **Oleaceae** | | | | | |
| 139 | *Forsythia suspensa* | Lian Qiao连翘 | Wild and cultivated | Thickets or grassy areas on slopes, valleys, gullies |  |
| 140 | *Ligustrum lucidum* | Nü Zhen Zi女贞子 | Wild and cultivated | Woods |  |
| 141 | *Osmanthus fragrans* | Gui Hua桂花 | Cultivated |  |  |
| **Orchidaceae** | | | | | |
| 142 | *Dendrobium officinale*, | Shi Hu石斛 | Wild and cultivated | Mountain, half-damp rock, warm and humid climate | CR, CITES II |
|  | *D. chrysanthum*, |  |  |  | VU, CITES II |
|  | *D. fimbriatum*, |  |  |  | VU, CITES II |
|  | *D. loddigesii*, |  |  |  | VU, CITES II |
|  | *D. nobile* |  |  |  | VU, CITES II |
| 143 | *Nervilia fordii* | Qing Tian Kui青天葵 | Wild | Shaded damp places in forests | CITES II |
| **Paeoniaceae** | | | | | |
| 144 | *Paeonia lactiflora*, | Chi Shao赤芍 | Wild and cultivated | Woods, grasslands | Regional Red Data Book |
|  | *P. veitchi* |  |  |  |  |
| 145 | *Paeonia sterniana* | Bai Shao白芍 | Wild and cultivated | Woods |  |
| 146 | *Paeonia suffruticosa* | Mu Dan Pi牡丹皮 | Wild and cultivated | Cliffs |  |
| **Palmae** | | | | | |
| 147 | *Areca catechu* | Da Fu Pi大腹皮 | Cultivated | Farming fields, homegardens |  |
| **Pandanaceae** | | | | | |
| 148 | *Pandanus tectorius* | Ye Bo Luo野菠萝 | Wild | Seashores, sandy beaches |  |
| ***Pedaliaceae*** | | | | | |
| 149 | *Sesamum indicum* | Hei Zhi Ma黑芝麻 | Cultivated |  |  |
| **Plantaginaceae** | | | | | |
| 150 | *Plantago asiatica* | Che Qian车前 | Wild | Mountain slopes, ravines, riverbanks, fields, roadsides, wastelands, lawns |  |
| **Poaceae** | | | | | |
| 151 | *Bambusa tuldoides*, | Zhu Ru竹茹 | Wild and cultivated | Low hills, river banks |  |
|  | *Dendrocalamus beecheyana* var. *pubescens*, |  |  |  |  |
|  | *Phyllostachys glauca* |  |  |  |  |
| 152 | *Coix lacryma-jobi* | Yi Yi Ren薏苡仁 | Cultivated | Dry fields, farming lands |  |
| 153 | *Imperata cylindrica* | Bai Mao Gen白茅根 | Wild and cultivated | River and seashore sands, disturbed grassy places |  |
| 154 | *Lophatherum gracile* | Dan Zhu Ye淡竹叶 | Wild | Shady slopes, roadsides and in moist forests |  |
| 155 | *Oryza sativa* | Jing Mi粳米 | Cultivated | Paddy fields |  |
| 156 | *Oryza sativa* var. *glutinosa* | Nuo Dao糯稻 | Cultivated | Paddy fields |  |
| 157 | *Phragmites australis* | Lu Wei芦苇 | Wild and cultivated | Moist places along river banks and lake margins, forming large colonies |  |
| 158 | *Pogonatherum crinitum* | Jin Si Cao金丝草 | Wild | Mountain slopes, forests, moist places along roadsides and streams |  |
| 159 | *Saccharum sinense* | Zhu Zhe竹蔗 | Cultivated | Farming lands |  |
| 160 | *Triticum aestivum* | Mai Ya麦芽 | Cultivated | Farming lands |  |
| 161 | *Zea mays* | Yu Mi Xu玉米须 | Cultivated | Farming lands |  |
| **Polygonaceae** | | | | | |
| 162 | *Fagopyrum dibotrys* | Jin Qiao Mai金荞麦 | Wild and cultivated | Moist valleys, grassy slopes |  |
| 163 | *Polygonum aviculare* | Bian Xu萹蓄 | Wild | Near fields, roadsides, waste places |  |
| 164 | *Polygonum chinense* | Huo Tan Mu火炭母 | Wild | Wet valleys, grassy slopes, mixed forests, thickets in valleys, mountain slopes |  |
| 165 | *Polygonum cuspidatum* | Hu Zhang虎杖 | Wild | Thickets in valleys, field margins, mountain slopes |  |
| 166 | *Polygonum hydropiper* | La Liao辣蓼 | Wild | Riverbanks, streamsides, wet valleys |  |
| 167 | *Fallopia multiflora* | He Shou Wu何首乌 | Wild | Mountain slopes, rock crevices, thickets in valleys |  |
| 168 | *Rheum palmatum* | Da Huang大黄 | Wild | Slopes, valleys |  |
| 169 | *Pyrrosia lingua* | Shi Wei石苇 | Wild | Stock, tree |  |
| 170 | *Wolfiporia extensa* | Fu Ling茯苓 | Wild | *Pinus densiflora* and *Pinus massoniana* forests |  |
| **Portulacaceae** | | | | | |
| 171 | *Portulaca oleracea* | Ma Chi Xian马齿苋 | Wild | Roadsides, farming fields |  |
| **Primulaceae** | | | | | |
| 172 | *Lysimachia christiniae* | Jin Qian Cao金钱草 | Wild | Damp areas along streamsides, open forests, forest margins |  |
| **Pteridaceae** | | | | | |
| 173 | *Doryopteris ludens* | Feng Wei Cao凤尾草 | Wild | Mountain forests, slopes |  |
| **Punicaceae** | | | | | |
| 174 | *Punica granatum* | Shi Liu石榴 | Cultivated | Orchards, homegardens |  |
| **Ranunculaceae** | | | | | |
| 175 | *Pulsatilla chinensis* | Bai Tou Weng白头翁 | Wild | Forest margins, slopes |  |
| 176 | *Cimicifuga foetida* | Sheng Ma升麻 | Wild | Forests, forest margins, grassy slopes, mountains |  |
| 177 | *Coptis chinensis* | Huang Lian黄连 | Wild and cultivated | Forests, shaded places in valleys | VU |
| **Rhamnaceae** | | | | | |
| 178 | *Berchemia lineata* | Tie Bao Jin铁包金 | Wild | Hills, open places, roadsides; low elevations |  |
| 179 | *Ziziphus jujuba* | Zao 枣 | Cultivated | Mountains, hills, sunny dry slopes, plains |  |
| **Rosaceae** | | | | | |
| 180 | *Agrimonia pilosa* | Long Ya Cao龙牙草 | Wild | Thinned forests, forest margins, thickets, meadows, stream banks, roadsides |  |
| 181 | *Chaenomeles sinensis*, | Mu Gua木瓜 | Cultivated | Vegetable gardens, homegardens |  |
|  | *C. speciosa* |  |  |  |  |
| 182 | *Crataegus pinnatifida* | Shan Zha山楂 | Wild and cultivated | Among shrubs, slopes |  |
| 183 | *Eriobotrya japonica* | Pi Pa Ye枇杷叶 | Cultivated | Orchards, homegardens |  |
| 184 | *Armeniaca vulgaris* | Xing Ren杏仁 | Cultivated | Orchards, homegardens |  |
| 185 | *Prunus mume* | Wu Mei乌梅 | Cultivated | Orchards, homegardens |  |
| 186 | *Prunus sibirica* | Shan Xing山杏 | Wild | Mountain slopes, roadsides |  |
| 187 | *Rosa laevigata* | Jin Ying Gen金樱根 | Wild | Thickets, scrub, open montane areas, open fields, farmland |  |
| 188 | *Rosa rugosa* | Mei Gui玫瑰 | Wild and cultivated | Coastal hillsides, sandy soils on sea shores, offshore islands |  |
| **Rubiaceae** | | | | | |
| 189 | *Gardenia jasminoides* | Zhi Zi栀子 | Wild and cultivated | Thickets and forests at streamsides, on mountain slopes or hills, or in valleys or fields |  |
| 190 | *Hedyotis diffusa* | Bai Hua She She Cao白花蛇舌草 | Wild | Paddy fields, ridges of farmlands, humid open fields |  |
| 191 | *Paederia scandens* | Ji Shi Teng鸡屎藤 | Wild | Roadsides |  |
| 192 | *Uncaria sinensis*, | Gou Teng钩藤 | Wild | Sparse forests or wet secondary forests at middle elevations |  |
|  | *U. macrophylla*, |  |  |  |  |
|  | *U. rhynchophylla* |  |  |  |  |
| **Rutaceae** | | | | | |
| 193 | *Citrus aurantium* | Zhi Shi枳实 | Cultivated | Orchards, homegardens |  |
| 194 | *Citrus maxima* | Ju Hong桔红 | Cultivated | Orchards, homegardens |  |
| 195 | *Citrus medica* | Xiang Yuan香橼 | Wild and cultivated | Orchards, homegardens |  |
| 196 | *Citrus reticulata* | Ju橘 | Cultivated | Hillside forests; low elevations |  |
| 197 | *Phellodendron amurense* | Huang Bo黄檗 | Cultivated | Mountain slopes |  |
| 198 | *Ruta graveolens* | Chou Cao臭草 | Wild | Moutain ridges, slopes |  |
| **Sabiaceae** | | | | | |
| 199 | *Sabia japonica* | Qing Feng Teng清风藤 | Wild | Dense forests, forest margins, mountains, valleys, roadsides |  |
| **Saururaceae** | | | | | |
| 200 | *Houttuynia cordata* | Yu Xing Cao鱼腥草 | Wild and cultivated | Ravines, streamsides, forests, wet meadows, slopes, thicket and field margins, trailsides, roadsides, ditch banks |  |
| **Schisandraceae** | | | | | |
| 201 | *Schisandra chinensis* | Wu Wei Zi五味子 | Wild and cultivated | Ravines, slopes, along rivers |  |
| **Scrophulariaceae** | | | | | |
| 202 | *Rehmannia glutinosa* | Sheng Di Huang生地黄 | Wild and cultivated | Mountain slopes, trailsides |  |
| 203 | *Scrophularia ningpoensis* | Xuan Shen玄参 | Wild | Bamboo forests, along streams, thickets, tall grasses |  |
| 204 | *Striga asiatica* | Du Jiao Jin独脚金 | Wild | Crop fields, grasslands |  |
| **Selaginellaceae** | | | | | |
| 205 | *Selaginella tamariscina* | Juan Bai卷柏 | Wild | Sunny dry rock seam |  |
| **Smilaceae** | | | | | |
| 206 | *Smilax china* | Ba Qia菝葜 | Wild | Forests, thickets, hillsides, grassy slopes, shaded places along valleys or streams |  |
| 207 | *Smilax glabra* | Tu Fu Ling土茯苓 | Wild | Forests, thickets, thinly forested slopes along valleys, river banks |  |
| **Solanaceae** | | | | | |
| 208 | *Lycium chinense* | Gou Qi枸杞 | Cultivated | Slopes, wastelands, saline places, roadsides, near houses |  |
| **Stemonaceae** | | | | | |
| 209 | *Stemona tuberosa* | Bai Bu百部 | Wild | Forest margins, thickets, mountain slopes, trail sides |  |
| **Sterculiaceae** | | | | | |
| 210 | *Helicteres angustifolia* | Shan Zhi Ma山芝麻 | Wild | Sloping grasslands |  |
| 211 | *Sterculia lychnophora* | Pang Da Hai胖大海 | Cultivated | Village sides |  |
| **Tiliaceae** | | | | | |
| 212 | *Microcos stauntoniana* | Bu Zha Ye布渣叶 | Wild | Forested lands |  |
| **Tremellaceae** | | | | | |
| 213 | *Tremella fuciformis* | Yin Er银耳 | Cultivated | Green houses or indoors with deadwood & sawdust |  |
| **Trilliaceae** | | | | | |
| 214 | *Paris polyphylla* var. *yunnanensis* | Dian Chong Lou滇重楼 | Wild | Broad-leaved or coniferous forests, bamboo forests, thickets, grassy slopes | EN |
| **Valerianaceae** | | | | | |
| 215 | *Patrinia scabiosifolia* | Bai Jiang Cao败酱草 | Wild | Forests, forest margins, thickets, grassy areas, roadsides |  |
| **Verbenaceae** | | | | | |
| 216 | *Clerodendrum chinense* | Chou Mo Li臭茉莉 | Wild | Roadsides, mountain slopes |  |
| 217 | *Vitex negundo* | Wu Zhi Gan五指柑 | Wild | 100-3200 m |  |
| **Zingiberaceae** | | | | | |
| 218 | *Alpinia officinarum* | Gao Liang Jiang高良姜 | Wild | Forests, mountain slopes |  |
| 219 | *Amomum testaceum* | Bai Kou Ren白蔻仁 | Wild | Forests |  |
| 220 | *Amomum villosum* | Sha Ren砂仁 | Cultivated | Forests, cultivated in wet and shady places in sparse forests |  |
| 221 | *Curcuma aromatica* | Yu Jin郁金 | Wild and cultivated | Mountain slopes, homegardens |  |
| 222 | *Zingiber officinalis* | Jiang姜 | Cultivated | Dry fields, vegetable gardens, homegardens |  |
